# U.S. Vietnamese Parents' Perceptions of Different Approaches for Confirming Adolescent HPV Vaccination Records in Research Studies

**DOI:** 10.1111/hex.70448

**Published:** 2025-09-29

**Authors:** Lilian Bui, Valerie Espinoza, Huyen‐Anh Giang, Linh P. Schiffer, Phuong Do, Phuong Ngu, Chi Vu, Jasmin Tiro, Eric Adjei Boakye, Siobhan M. Phillips, Heather Brandt, Ella Berry, Sa Tran, Emma Macdonald, Cindy Khuc, Jeanette Nguyen, Thy Vuong, Milkie Vu

**Affiliations:** ^1^ Department of Preventive Medicine, Feinberg School of Medicine Northwestern University Chicago Illinois USA; ^2^ Department of Public Health Sciences The University of Chicago Chicago Illinois USA; ^3^ Center to Eliminate Cancer Inequity The University of Chicago Chicago Illinois USA; ^4^ Comprehensive Cancer Center University of Chicago Medicine Chicago USA; ^5^ Department of Public Health Sciences Henry Ford Health System Detroit Michigan USA; ^6^ Department of Otolaryngology – Head and Neck Surgery Henry Ford Health System Detroit Michigan USA; ^7^ Henry Ford Health + Michigan State University Health Sciences Detroit Michigan USA; ^8^ Robert H. Lurie Comprehensive Cancer Center Northwestern University Chicago Illinois USA; ^9^ Department of Epidemiology and Cancer Control St. Jude Children's Research Hospital Memphis Tennessee USA; ^10^ HPV Cancer Prevention Program St. Jude Children's Research Hospital Memphis Tennessee USA; ^11^ Chinese Mutual Aid Association Chicago Illinois USA; ^12^ Rush University College of Nursing Chicago Illinois USA; ^13^ Canberra Health Services Garran ACT Australia

**Keywords:** Asian Americans, digital health, HPV vaccine, patient engagement, U.S. Vietnamese, vaccination status

## Abstract

**Background:**

The United States (U.S.) Vietnamese communities face a high burden of HPV‐related cancer rates; they also have low HPV vaccination coverage. HPV vaccination is a safe, effective tool to prevent HPV‐related cancers, particularly when administered during adolescence. Understanding Vietnamese parents' perspectives on the acceptability of different HPV vaccination status verification methods can improve the implementation of community‐based research interventions to improve HPV vaccine coverage in this population.

**Objective:**

We assessed their perceptions of three HPV vaccination status confirmation methods: medical records, vaccination card photographs, and self‐reported surveys.

**Design:**

We conducted interviews with Vietnamese parents of adolescents ages 9–18, healthcare providers, and community leaders (*n* = 34). We used an inductive thematic analysis approach with four qualitative coders.

**Results:**

Preferences for confirming vaccination status were split almost equally among each method. Key drivers of these preferences included privacy concerns, autonomy, efforts required from parents, digital literacy, and data accuracy.

**Discussion:**

These findings suggest offering multiple confirmation options to accommodate diverse preferences and enhance the effectiveness of HPV vaccination promotion strategies in this population.

**Public Contribution:**

Community involvement was integral to the design, implementation of this project, and writing of the manuscript. The study team partnered with a community organization that works closely with the U.S. Vietnamese population and Vietnamese healthcare providers. The group met regularly to discuss participant recruitment strategies and study instruments. Lived experiences from parents of adolescents, community leaders, and healthcare providers were included in the production of this manuscript to guide the interpretation of preferences for vaccination confirmation methods.

## 1 Introduction

HPV vaccination is a safe and effective method to prevent HPV‐related cancers, including anal, cervical, oropharyngeal, penile, vaginal, and vulvar cancers [1, 2, 3]. While it can be given starting at age 9 and up to the age of 45, the vaccine is the most effective when administered to adolescents [4]. Despite its benefits, HPV vaccine coverage among adolescents remains suboptimal in the United States (U.S.) and is below the *Healthy People 2030* target of 80% [5]. Moreover, many populations in the U.S. experience disparities in HPV vaccine coverage. In particular, the U.S. Vietnamese community has disproportionately higher cervical cancer incidence and lower HPV vaccination rates (52% initiation and 35% completion compared to national rates for all adolescents of 75% initiation and 59% completion), which underscores the need for targeted intervention efforts [6, 7].

Assessment of HPV vaccination status is critical for identifying disparities in coverage and evaluating the effectiveness of vaccine promotion efforts [8, 9, 10]. Outside of the clinical setting, research studies with participants typically use three approaches to assess HPV vaccination status. These approaches involve research participants either (i) consenting for researchers to request vaccination records directly from participants' healthcare providers or immunization registries [11, 12, 13], (ii) submitting their HPV vaccination cards or medical records to the research team [14, 15], or (iii) self‐reporting vaccination status and dates in a survey format [16, 17, 18].

Among studies focusing on HPV vaccination in Asian American populations, self‐report has been the most used method. A systematic review found that 16 out of 26 studies assessing HPV vaccination among Asian Americans relied on self‐reported data [19]. While self‐report is a widely used method, it can be subject to recall and social desirability bias and result in misclassification of data and/or misdirected intervention efforts for a population. To our knowledge, only one study on HPV vaccination in the Asian American populations has used medical records to confirm HPV vaccination status of research participants [20]. Medical records provide objective and verifiable measures; however, they may also raise privacy concerns among research participants [21]. A study evaluating Hepatitis B vaccination in Chinese, Korean, and Vietnamese populations collected both self‐reports and vaccination cards and found high agreement between the two methods [22].

Some previous research has focused on comparing the accuracy of self‐reports of vaccination to medical records in different populations [8, 9, 10, 23, 24]. However, no research has focused on how U.S. Vietnamese parents view these HPV vaccination status verification methods, and which may be most acceptable and feasible for this population. Gaining such community input will improve the implementation of community‐based research interventions to improve HPV vaccine coverage, particularly through increasing reach and broadening participant diversity. This study explored the perceived advantages, disadvantages, and preferences of U.S. Vietnamese parents regarding various vaccination status verification methods.

## 2 Materials & Methods

### 2.1 Design and Setting

Data for this study came from Project HERO (HPV Education and Resources for the U.S. Vietnamese Population), a larger initiative that aimed to develop and test a culturally‐relevant digital health intervention to improve U.S. Vietnamese caregivers and parents' uptake of the HPV vaccine for their adolescents. This study represents the initial phase, which took place between February and November 2024, and focuses on identifying barriers and facilitators to enrollment in digital health interventions for HPV vaccination. Participants for this study were recruited using a combination of convenience and snowball sampling in collaboration with community‐based organizations (CBOs) in the Chicagoland area. Recruitment efforts leveraged outreach at community events and programs hosted by these CBOs. In addition, we also used online recruitment strategies to expand reach within the target population. Participants were eligible if they: (1) have lived in the U.S. for ≥ 12 months; and (2) were either: a U.S. Vietnamese parent of an individual aged 9 to18, a healthcare provider working with the U.S. Vietnamese population, and/or a community leader working with the U.S. Vietnamese population. Participants first completed an online survey assessing sociodemographic characteristics, digital health literacy, and knowledge of HPV and the HPV vaccine. They then took part in a semi‐structured interview ( ~ 45 min). Both the surveys and interviews were offered in either English or Vietnamese based on participants' preferences. Each participant received a $50 gift card as compensation. The Northwestern University Institutional Review Board approved of this study (STU00219808).

### 2.2 Survey Measurements

We assessed participants' knowledge of HPV and the HPV vaccine using items from the Human Papillomavirus (HPV)‐Knowledge 18 [25]. While not yet validated in the Vietnamese language and/or among the U.S. Vietnamese population, this questionnaire has been previously validated in a sample of adults (*n* = 256) in the U.S. The scale included 18 true/false items assessing participants' knowledge of HPV. Examples include: “There is a screening that is commonly used to test males for HPV” (false), “HPV can cause penile cancer” (true), “Certain types of HPV can lead to cervical cancer in women” (true), and “Nearly all sexually active men and women will contract HPV at some point” (true). Participants received a score of 1 for each correct response and 0 for incorrect or “I do not know” responses. The total HPV knowledge score was calculated as the sum of correct responses, with higher scores indicating greater knowledge. In our sample, the Cronbach's alpha for the scale was 0.87.

In addition, we assessed participants' digital health literacy using nine items from the Digital Health Literacy Instrument [26]. Participants were asked: “When you search the Internet for information on health, how easy or difficult is it for you to…” Example items included: “Decide whether the information is reliable or not?” and “Use the information you found to make decisions about your health (e.g., on nutrition, medication, or whether to seek a doctor's opinion)?” Participants rated each item on a four‐point scale: Very difficult, Somewhat difficult, Somewhat easy, and Extremely easy. In our sample, the Cronbach's alpha for the scale was 0.83.

Moreover, we assessed several sociodemographic characteristics, including age, gender, country of birth, length of time in the U.S., education, and ability to understand medical information in English and Vietnamese.

### 2.3 Semi‐Structured Interviews

The interview guide was developed based on a review of relevant literature and in consultation with staff members from our partner CBOs. Participants were asked about their usage of and attitudes towards digital health tools as well as their preferences for methods of vaccination status verification. Examples of interview questions included, but were not limited to:

– Some researchers ask parents for permission to obtain vaccination records from the child's doctor. After the parents give the researchers permission, the researchers would then contact the doctor's office directly. What do you think about this method? Benefits? Any downsides? Would you be willing to give the researcher permission to obtain your child's vaccination records from the doctor?
– Some researchers ask parents to take a picture of the vaccination card or records and send it to the researchers. What do you think about this method? Benefits? Any downsides? Would you be willing to share a picture of the vaccination card or records with the researchers?
– Some researchers ask parents to self‐report vaccination via a survey. Parents would fill out this survey and indicate whether and when they have gotten the vaccine. What do you think about this method? Benefits? Any downsides? Would you be willing to self‐report vaccination via a survey?

### 2.4 Data Analysis

All statistical analyses were conducted in SPSS version 29.0.2.0 (“IBM SPSS Statistics for Windows” 2023) [27]. Descriptive statistics were used to summarize sociodemographic information, knowledge of HPV and the HPV vaccine, and digital health literacy. Chi‐square and two‐tailed *t*‐tests were conducted to examine potential differences in preferences based on gender, length of time in the U.S., education, language fluency, knowledge of HPV and the HPV vaccine, and digital health literacy. All interviews were recorded and transcribed verbatim. The Vietnamese transcripts (25 out of 34) were then translated into English by a bilingual team member. For each translation, three additional bilingual team members reviewed and validated it to ensure accuracy. All interview data were analyzed using MAXQDA 24 [28]. We used an inductive thematic analysis approach [29, 30]. First, research team members independently reviewed around half of the transcripts [31], created memos, generated qualitative codes using open, axial, and selective coding [30, 32, 33], and created a codebook with code definitions, examples, inclusion, and exclusion criteria [30]. Then, using the codebook, four coders independently recoded all transcripts, constantly comparing results and resolving any discrepancies through team meetings. We then organized the coded data to develop initial themes and subthemes. We used researcher triangulation, as well as vetted themes and subthemes by research team members and reached a consensus on themes [34]. Study researchers finalized all themes through discussion. To establish and enhance validity, rigor, and trustworthiness in qualitative research, we employed several techniques, including articulating data collection and analysis decisions, providing verbatim transcription, exploring rival explanations, and close collaboration with community partners [35].

## 3 Results

### 3.1 Quantitative Results

We interviewed 34 participants, some of whom held multiple roles. Eight participants identified as community leaders. Among the 22 parents who identified primarily as U.S. Vietnamese parents of adolescent children, five of them also identified as community leaders. Additionally, four participants identified primarily as healthcare providers; among those, one also identified as a community leader. Table 1 displays their sociodemographic characteristics. The majority of participants were female (88.2%), were born in Vietnam (82.4%), and reported limited ability to understand medical information in English (70.6%). The mean score on the HPV‐Knowledge 18 measuring knowledge of HPV and the HPV vaccine was 8.29 (SD = 4.66, range 0–18). Regarding digital health literacy, many participants reported challenges in navigating online health information. The most commonly cited difficulty was determining whether information was written with a commercial interest (79.4%), followed by assessing the reliability of information found online (70.6%). Moreover, half of the participants (50.0%) struggled to find the exact information they were looking for.

**Table 1:** Sociodemographic characteristics, knowledge of HPV and the HPV vaccine, and digital health literacy.

Characteristic	N (%) or M (SD)
Gender	
Male	4 (11.8%)
Female	30 (88.2%)
Age	
25–34 years old	6 (17.6%)
35–44 years old	15 (44.1%)
45–54 years old	12 (35.3%)
55–64 years old	1 (2.9%)
Country of birth	
Vietnam	28 (82.4%)
Another country	6 (17.6%)
Length of time in the U.S.	
2–10 years	13 (38.2%)
11–20 years	9 (26.5%)
More than 20 years	12 (35.3%)
Education	
High school or less	10 (29.4%)
Bachelor's degree	11 (32.4%)
Advanced degree	13 (38.2%)
Difficulty in understanding medical information in English	24 (70.6%)
HPV‐Knowledge 18 score (range 0–18)	8.29 (4.66)
Item least correctly answered: *“There is a screening that is commonly used to test males for HPV.”*	4 (11.8%)
Item most correctly answered: *“Certain types of HPV can lead to cervical cancer in women.”*	28 (82.4%)
Digital health literacy	
Difficulty in making a choice from all the information found	14 (41.2%)
Difficulty in using proper words or search query to find information	12 (35.3%)
Difficulty in finding exact information being looked for	17 (50.0%)
Difficulty in deciding whether information is reliable	24 (70.6%)
Difficulty in deciding whether information is written with commercial interest	27 (79.4%)
Difficulty in checking different websites to see if they provide same information	16 (47.1%)
Difficulty in deciding if information found is applicable	14 (41.2%)
Difficulty in applying the information in daily life	11 (32.4%)
Difficulty in using information found to make decisions about health	11 (32.4%)

For the most part, we did not find differences in preferences for confirming vaccination status based on gender, length of time in the U.S., education, language fluency, knowledge of HPV and the HPV vaccine, and digital health literacy. Those who indicated a preference for sending in vaccination cards appeared to have a higher HPV knowledge score (M = 10.08, SD = 3.34) than those who did not indicate this preference (M = 7.32, SD = 5.05), but these differences did not reach statistical significance (*p* = 0.10; supplemental analyses not shown in tables).

### 3.2 Qualitative Results

Preferences for confirming vaccination status were split almost equally among each method. In the sample, 32.3% preferred consenting for researchers to access medical records, 35.3% preferred sending in vaccination cards, and 32.3% preferred self‐reported surveys. Key drivers of these preferences included *privacy concerns, autonomy, efforts required from parents, digital literacy, and data accuracy* (Tables 2 and 3).

**Table 2:** Themes related to participants’ perceptions of verification methods and examples of quotes.

Themes	Quotes
Consent for researchers to access participants' medical records	
Advantages	
Data accuracy	“You'll get the most information and the most accurate information if you can get parents to consent to reaching out to healthcare providers versus having parents self‐report.” (Community leader) “It is more accurate in terms of you would know for sure, because sometimes parent even the good ones, get their kids to take every vaccination possible, but they don't remember everything, right? Because there are so many different types of vaccination. It is more accurate that way.” (Parent, also a community leader)
Less effort required from parents	“I think it's the least amount of work for the parent, and that seems like a barrier to me when you tell the parents that you want them to be involved in something, and then there's one thing, and then another thing, and then another thing.” (Community leader) “That is an easy method as long as the parent gives consent, because then the parent doesn't have to worry about filling out something online, filling out something on a piece of paper and then sending it, sending it to you or your team.” (Community leader) “Getting that information is probably easy because parents don't have to do anything.” (Parent)
Disadvantages	
Privacy concerns	“In the U.S., everything asks for your permission. People also have the idea that it's too much when your information is being bought and sold, phone number, web browsing history, or how we use our phones… stuff like that. So, I feel like I don't like it. It doesn't ensure my privacy. When something requires my permission, it's highly likely that I will say no.” (Parent) “They are afraid that their children's information would be leaked because they don't know what the researchers are capable of doing with this information.” (Parent) “If necessary, I will give you permission. But I don't think others would, the people here don't like things that involve their personal information.” (Parent, also a community leader) I think just generally, there is fear about releasing information and sharing information, seeing how sensitive immigration status can be especially amongst parents. (Community leader)
More effort required from providers	“Downside as far as… involves doctors cooperating.” (Healthcare provider) “That method is good, but it will bother the doctors of each child. It takes time to find each child's doctor.” (Parent) “It's just extra work from my staff. I'm just thinking selfishly. But yeah, in the name of research, everyone has to sweat, right?” (Healthcare provider)
Prerequisite	
Necessary to establish trust	“If I trust you and I feel that your mission is very good, there will be no problem with me at all. I will give you the permission so that you can obtain my child's vaccination record.” (Parent, also a community leader) “If you can show me that this program is to help the Vietnamese community increase their knowledge about vaccines, I will trust you and really want this.” (Parent, also a community leader) “Since this is a research project beneficial towards society, I would agree. But there are also people who don't know you and what HPV is, they will see this as information getting leaked out. Especially in the U.S. where privacy is very important, people may not have enough confidence to provide their information like that.” (Parent, also a community leader)
Vaccination card	
Advantages	
Less effort required from parents with digital literacy	“I would rather take a photo. I would prefer the second method, it's quick because I don't have to do anything… I would just need to take a picture and send it, and I won't have to fill out anything. It'll be quicker…” (Parent, also a community leader) “I mean, I think that would be easier. Since then, the parents can just text it to you. And then, you already have a phone number and name confirmed to it.” (Community leader) “I think they're all the same, but this one has a benefit in that you're directly contacting this person, and they provide it to you immediately. With the first method, you have to take an extra step to go see the doctor. While that might take some time, this method is very convenient, which means that you can receive it directly and immediately.” (Parent) “Many parents keep their children's vaccination records from their doctors so they can submit them to the school.” (Community leader) “That might be a slightly better method, because I have worked with a lot of parents who, like as I mentioned, we help them with a lot of applying for jobs, applying for licenses, passports, all these things, and they always have like pictures of IDs, social security cards, any sort of records. I think that would be a pretty good method to use.” (Community leader)
Promotes autonomy	“I think it's safer than the other one that asks for permission. I feel like I don't know if they'll use my vaccination information for anything other than HPV. I don't like giving permission to access everything. But if you need proof of vaccination, I can do it and send it to you.” (Parent)
Disadvantages	
Privacy concerns	“If it covers the name, then okay, fine. The second method is the vaccination list, but if it covers the name, then I'll give it to you. But I won't allow it if the child's name is on it.” (Parent) “Yes, I can send you a photo of the vaccination card. I would cover my child's name, age, and date of birth on the card.” (Parent)
More effort required from parents without digital literacy	“If I don't have it on hand, it will take a lot of effort to find it and to know where it is. Because, like me right now, I rarely log in to those accounts, so I also forgot about them.” (Parent) “Sometimes it's hard to for certain families to obtain certain documents and things like that, and that might include like vaccination or doctors' records.” (Community leader) “Many parents don't even know where they should get vaccination cards.” (Parent, also a community leader) “It's a little more work for me. I have to go to the hospital to get the record, take a photo, and then send it away, it's a little more work.” (Parent) “I think that's too bothersome. I don't want to do it since it's not necessary.” (Parent, also a community leader)
Self‐reported surveys	
Advantages	
Less efforts required from parents	“Well, it's fast, isn't it? It's quick to answer. I don't lose anything, I'm not impacted by it, and I don't have to give permission. I think it's the most convenient.” (Parent) “Benefits of self‐reporting? You're gonna get the data done quicker opposed to trying to go around…You're using more resources to try to figure out where these people are, where their records are, trying to follow up with them to get the pictures, or the copy of the records. You're gonna have everything you need in front of you and the parents are gonna have everything you want from them in front of them. You don't have to come follow back up like, “Are you sure you're self‐reporting?” The correct thing is “Yes, self‐reported or no”, keep it simple.” (Community leader)
Promotes autonomy	“Because they might not want to show the records but for this method, they just need to fill in a form.” (Parent) “I think this is also good, since I know and I'm filling it out myself, it's more okay.” (Parent) “So self‐reporting is beneficial in that way, they can choose what information they feel comfortable sharing.” (Community leader)
Disadvantages	
Data inaccuracy	“Parents will not remember if they are asked about the date and the month of vaccinations, they would have to check again. If you only ask about the HPV vaccine, it may be ok, but if you need a list of all other vaccines, it is probably difficult. It is even more difficult for parents with two or three children.” (Parent, also a community leader) “People and their HPV vaccination, especially for Vietnamese patients, they don't remember they had the vaccine.” (Healthcare provider) “But then you also have to take into account that they may not be truthful on their answers, if they want for whatever reason to communicate or show that they did give their child the vaccine, but maybe they didn't.” (Community leader)
General concerns	
Fear of scammers and privacy concerns	“I think we're starting to develop a fear of what's being shared online. So, I think sharing information online, especially, I feel that we see a lot of misinformation on social media and parents and kids are becoming more like literate when it comes to knowing what's real and what's not, for parents it could be a fear like, ‘Is this real information?’, ‘Is this a scam?’, ‘Is this real?’, ‘Am I going to get scammed?’” (Community leader) “They are worried of being scammed.” (Parent, also a community leader) “But there are also people who don't know you and what HPV is, they will see this as leaking out information. Especially in the U.S. where privacy is very important, people may not find this reliable enough to provide their information like that.” (Parent, also a community leader) “There are people who won't want to because they don't trust whether you're going to take that record to do something else.” (Parent)

**Table 3:** Themes related to participants' comparisons of verification methods and examples of quotes.

Comparison of vaccination status confirmation methods	
Access to medical records versus other methods	“I think [access to medical records] is faster. [Photo of a vaccination card] takes more time.” (Healthcare provider) “I think it's [access to medical records]. Because the other ways require people to do it themselves, and they don't have time.” (Healthcare provider) “[Regarding photo of a vaccination card], you are just relying on the parent to complete that step, so they may forget, they may lose the piece of paper that they'd be sending the picture to. So, I think that just creates a larger opportunity for human error, whereas if you're calling the office directly, you'll definitely get the information that you requested, as long as the consent is there.” (Community leader)
Vaccination card versus other methods	“With [access to medical records], you have to take an extra step to go see the doctor. While [access to medical records] might take some time, [photo of a vaccination card] is very convenient, which means that you can receive it directly and immediately.” (Parent) “They can decide on this by themselves. They might have to ask the doctors for [access to medical records], if the doctors say no, then they can't get it. For [photo of a vaccination card], they would have the record at home. They can take pictures. If they trust the researchers, they will give it.” (Parent) “It's just hard to remember. They have to find the record and I don't see it being as easy for you as researchers to get the information you want, compared to the [access to medical records].” (Community leader)
Self‐reported surveys versus other methods	“They might be more comfortable with [self‐reports]. Because they might not want to show the [medical] records but for [self‐reports], they just need to fill in a form.” (Parent) “Self‐reporting doesn't involve… that same privacy issue as there is with sending a picture of a document or getting consent to talk to the doctor or physician. So self‐reporting is beneficial in that way, they can choose what information they feel comfortable sharing.” (Community leader)

#### 3.2.1 Medical Records and Photos of Vaccination Cards Can Provide Higher Data Accuracy, but Raise Privacy Concerns

When asked about consent for researchers to access vaccination records directly from healthcare providers or immunization registries, or if participants would be willing to send a photo of their children's vaccination cards, most participants agreed that the main benefit of these methods is improved data accuracy. A participant compared the methods: “[Regarding photo of a vaccination card], you are just relying on the parent to complete that step, so they may forget, they may lose the piece of paper that they'd be sending the picture to. So, I think that just creates a larger opportunity for human error, whereas if you're calling the office directly, you'll definitely get the information that you requested, as long as the consent is there.” Some participants thought that this method would also take less time from participants.

However, many also discussed their privacy concerns and reluctance to agree to these methods, particularly in light of past experiences with scams and fear of their personal information being leaked. For example, one participant noted that “When something requires my permission, it's highly likely that I will say no.” Similarly, another participant highlighted a general reluctance within the Vietnamese community to share personal data: “People here don't like things that involve their personal information.” Participants mentioned that community members were increasingly cautious about sharing information online, as they worried about being targeted for scams. One participant said, “For parents, it could be a fear like… ‘Am I going to get scammed?’”

Some participants suggested that their willingness to consent to medical records access or share photos of vaccination records depended on specific conditions. For instance, participants shared that they would be willing to send a photo of a vaccination card only if they could redact their child's name, age, and date of birth. Removing identifiable information would provide a sense of control over privacy risks. In addition, participants also said their willingness to consent to access to medical records depended on whether they trusted the research team or believed in the project's goal. A participant said: “If you can show me that this program is to help the Vietnamese community increase their knowledge about vaccines, I will trust you and really want this.”

#### 3.2.2 Photos of Vaccination Cards Can be Convenient and Promote a Sense of Autonomy but Present Challenges for Those With Low Digital Literacy Skills

To many participants, sending in a photo of the vaccination cards required minimal effort for those with digital literacy skills. Participants suggested that parents already kept photos of vaccination records for schools or other purposes and thought this approach would help streamline data collection. One participant said: “With [access to medical records], you have to take an extra step to go see the doctor. While [access to medical records] might take some time, [photo of a vaccination card] is very convenient, which means that you can receive it directly and immediately.”

Participants also felt that sending a photo promoted a sense of autonomy. Unlike consenting to researchers accessing medical records, participants felt like they could retain control over what information to share. One participant reflected on the differences between these methods, “I think it's safer than the other one that asks for permission. I feel like I don't know if they'll use my vaccination information for anything other than HPV. I don't like giving permission to access everything. But if you need proof of vaccination, I can do it and send it to you.”

However, participants also noted that this method could be challenging for parents who lacked digital literacy skills. A participant said: “If I don't have it on hand, it will take a lot of effort to find it and to know where it is. Because, like me right now, I rarely log in to those accounts, so I also forgot about them.” Another participant echoed: “Many parents don't even know where they should get vaccination cards.”

#### 3.2.3 Self‐Reported Surveys Require Less Effort and Promote Autonomy but May Compromise Data Accuracy

Regarding self‐reported surveys, some participants endorsed this method for its simplicity and low effort. Moreover, this method promotes parental autonomy, as it allows parents to independently provide information without sending in official documentation. One participant explained, “Because they might not want to show the records, but for this method, they just need to fill in a form.” Another participant compared this method to others: “Self‐reporting doesn't involve… that same privacy issue as there is with sending a picture of a document or getting consent to talk to the doctor or physician. So self‐reporting is beneficial in that way, they can choose what information they feel comfortable sharing.”

Nonetheless, participants raised concerns about the accuracy of self‐reported data. A participant explained, “People and their HPV vaccination, especially for Vietnamese patients, they don't remember they had the vaccine.” Another participant highlighted the potential for inaccuracies in self‐reported data due to social desirability bias, adding, “But then you also have to take into account that they may not be truthful on their answers… maybe they didn't [vaccinate their child] but want to communicate that they did.”

## 4 Discussion

This study highlights the importance of offering multiple methods for confirming HPV vaccination status in research studies to accommodate the diverse preferences and needs of U.S. Vietnamese parents. Participants prioritized factors such as privacy, autonomy, effort required from parents, digital literacy, and data accuracy. Notably, no single method emerged as universally ideal. Access to medical records was perceived as the most accurate and required minimal parental effort, but they raised significant privacy concerns. In contrast, vaccination card photos and self‐reported surveys promoted autonomy, though they came with trade‐offs, such as increased parental effort, challenges for those without digital literacy skills, and potential issues with data inaccuracy.

Concerns about the accuracy of self‐reports were common among participants in our study and are supported by findings from a few previous studies. A paper analyzing the National Immunization Survey – Teen data demonstrated that approximately one quarter of U.S. parents inaccurately reported HPV vaccination initiation and completion [24]. Another study evaluating the concordance between electronic medical records and self‐reports of receipt of eight adult vaccines reported that agreement statistics varied widely for each vaccine, though higher agreement was noted for the HPV vaccine [36]. In addition, a study found that up to 28.6% of children with high‐risk medical conditions had no documented influenza vaccination despite parental self‐reports of vaccination [37]. These findings validate concerns about the potential inaccuracy of self‐reported surveys. However, the convenience and perceived autonomy that come with self‐reports may improve participants' willingness to enroll in studies, which underscores the trade‐offs researchers must consider when selecting vaccination status confirmation methods.

Some existing literature has compared the reliability of the three methods explored in this study. Medical records are typically considered the gold standard against which vaccination cards and self‐reports are compared [38]. However, for HPV vaccination specifically, medical records may be an incomplete method of verification, due to missing records for vaccination (e.g., when people move from one health system to another, or if they are seeing multiple providers in different systems) and challenges with provider cooperation. For instance, a study using a statewide surveillance system found that available medical records did not contain information on HPV vaccination for 34% of the women in the study [39]. The same study found that when both medical records and self‐reported data were used, concordance was high (83%) [39].

In the 2023 National Immunization Survey—Teen, only 52.0% of the households consented to contact their vaccination providers, which indicated a high refusal rate for access to medical records [40]. Other research has shown that the concordance between medical records and self‐reports tends to be higher for the initiation of the HPV vaccine series (e.g., having received at least one dose) than for vaccine series completion or number of doses received [41, 42, 43]. Moreover, a study comparing adult proxy recall (i.e., self‐reports) with household immunization records (e.g., vaccination cards) found that adult proxy recall has a higher rate of sensitivity (84%), whereas household immunization records have a high rate of specificity (98%) when it comes to HPV vaccination [44].

Privacy concerns emerged as another consideration influencing participants' preferences for vaccination confirmation methods. A recurrent theme is the fear of scammers and the potential for personal information to be leaked. Immigrants, including those in the U.S. Vietnamese community, are frequently targeted by scams [45, 46, 47]. These concerns are further exacerbated by the evolving nature of cybercrimes and the vulnerabilities associated with immigration status and socioeconomic conditions, all of which make participants cautious about sharing personal data [46]. Additionally, low digital literacy further impacts privacy concerns and willingness to share personal data for research. Low digital health literacy can limit access to online patient portals [48] and hinder parents' ability to share vaccination cards with researchers. In our sample, digital health literacy scores were lower than those reported in other studies using the Digital Health Literacy Instrument [26, 49]. Similarly, participants' HPV‐Knowledge 18 scores were lower than those reported in the original validation study [25]. To our knowledge, these instruments have not yet been widely applied among immigrant or minority populations in the U.S., limiting our ability to make direct comparisons across these groups.

Our interviews revealed that trust often plays a pivotal role in participants' willingness to share personal information. Participants emphasized the importance of trust in the research team, understanding of how data would be used, and perceived direct benefits to their community. Although limited research has explored this issue among Asian American and racially and ethnically minoritized populations, prior studies offer useful insights. For instance, a study found that veterans' opinions of consent processes were closely tied to their trust in the Veterans Affairs organization [50]. Similarly, a study involving research participants from three academic health centers reported that they weighed the risks of data sharing against the potential benefits of research. Many participants were willing to share data if they better understood its intended use [51]. Systematic reviews also highlight the influences of trust in the organization or research team, concerns of data misuse, lack of knowledge of existing privacy safeguards, and perceived research benefit to society [52, 53]. Taken together, these findings underscore the significance of fostering trust, promoting digital literacy, and clearly articulating research benefits as strategies to mitigate reluctance in data sharing and encourage participation among underserved communities.

The findings highlighted the importance of offering multiple methods to confirm vaccination status in community‐based research with minoritized populations. The benefits include demonstrating a respect for participants' autonomy and reducing barriers to enrollment and data collection. Each method—medical records access, vaccination card submission, and self‐reported surveys—presents trade‐offs between data accuracy, privacy concerns, digital literacy challenges, and participant burden. Notably, the National Immunization Survey—Teen, often considered the gold standard for establishing HPV vaccination coverage in the U.S., asks for both self‐reports from parents as well as consent for accessing medical records [40], which highlights the importance of offering multiple verification methods. One consideration for future research is the approach by which multiple verification methods could be aggregated to estimate overall immunization coverage. Researchers could leverage medical records for higher specificity, while also using vaccination cards as secondary confirmation and self‐reports to reduce missingness. In future studies, it is important to continue exploring and comparing how coverage estimates may vary under different combinations of data sources.

Moreover, transparent communication about the purpose of data collection and data protection measures can increase trust among potential participants. One possible strategy is to partner with trusted community organizations and leaders, who could enhance the credibility of researchers and encourage community members to participate in research studies. Additionally, incorporating multimedia elements, such as videos explaining the study's purpose and consent process, alongside traditional text‐based consent forms, may improve comprehension and engagement, particularly for those with lower health or digital literacy [54, 55, 56]. By aligning research protocols with community preferences, researchers can better meet different needs and improve engagement in interventions.

### 4.1 Strengths and Limitations

Our study has several strengths. We conducted semi‐structured interviews in both Vietnamese and English, which allowed for more inclusive participation and enhanced the richness of data collection. Transcript translations were also validated by bilingual research team members. Moreover, we recruited not only parents but also community leaders and healthcare providers, which enabled us to collect more diverse perspectives and gain a more comprehensive understanding of the topic.

Nevertheless, the study has limitations. Given that we relied on convenience sampling, the potential for sampling bias exists (e.g., we may have recruited participants of similar backgrounds and perspectives rather than a random representation of the population). Given its qualitative design, our study may also have limitations in the transferability of findings to other contexts and settings [57]. Because the primary aim of this study was to conduct qualitative research to explore preferences and considerations around vaccination confirmation methods, it was not designed or powered to detect statistical associations between participant characteristics (e.g., HPV knowledge, digital health literacy) and their preferences. While exploratory analyses suggested a possible trend toward higher HPV knowledge among those preferring vaccination card submission, these findings did not reach statistical significance. Larger, quantitatively focused studies would be needed to rigorously examine such associations.

The HPV‐Knowledge 18 and Digital Health Literacy Instrument were translated from English into Vietnamese by bilingual team members, with independent verification by additional bilingual reviewers. A formal back‐translation procedure was not conducted. This streamlined approach allowed us to efficiently prepare linguistically accurate instruments for use in a small sample, but it does not provide the same level of rigor as full forward–back translation procedures.

## 5 Conclusion

Our study focusing on the U.S. Vietnamese community highlights the need for multiple methods to confirm HPV vaccination status. Our findings reveal that no single method is universally preferred; while giving access to medical records is perceived as the most accurate, it also raises significant privacy concerns. In contrast, vaccination card photos and self‐reported surveys promote autonomy but come with challenges such as increased parental effort and potential data inaccuracy. Privacy concerns, particularly related to online scams and data security, emerged as critical factors shaping participants' willingness to share vaccination information. Trust in the research team and a clear understanding of how data would be used played a key role in participants' willingness to engage in research. These trade‐offs highlight the need for culturally sensitive and flexible approaches to data collection in community‐based research.

## Author Contributions

**Lilian Bui:** methodology, formal analysis, validation, writing – original draft, writing – review and editing. **Valerie Espinoza:** methodology, formal analysis, validation, writing – review and editing. **Huyen‐Anh Giang:** methodology, formal analysis, validation, writing – review and editing. **Linh P. Schiffer:** methodology, formal analysis, validation, writing – review and editing. **Phuong Do:** methodology, formal analysis, validation, writing – review and editing. **Phuong Ngu:** methodology, formal analysis, validation, writing – review and editing. **Chi Vu:** methodology, formal analysis, validation, writing – review and editing. **Cindy Khuc:** methodology, formal analysis, validation, writing – review and editing. **Jeanette Nguyen:** methodology, formal analysis, validation, writing – review and editing. **Jasmin Tiro:** writing – review and editing. **Eric Adjei Boakye:** writing – review and editing. **Siobhan Phillips:** writing – review and editing. **Heather Brandt:** writing – review and editing. **Thy Vuong:** writing – review and editing. **Ella Berry:** project administration, writing – review and editing. **Sa Tran:** project administration, writing – review and editing. **Emma Macdonald:** project administration, writing – review and editing. **Milkie Vu:** methodology, formal analysis, data curation, funding acquisition, project administration, writing – original draft, writing – review and editing, supervision (corresponding and submitting author). All authors contributed to manuscript preparation and approved the final version.

## Disclosure

The funders played no role in the design and conduct of the study; collection, management, analysis, or interpretation of the data; preparation, review, or approval of the manuscript; and decision to submit the manuscript for publication.

## Ethics Statement

The Northwestern University Institutional Review Board approved of this study (STU00219808).

## Conflicts of Interest

The authors declare no conflicts of interest.

## Data Availability

The data that support the findings of this study are available from the corresponding author, Milkie Vu, upon reasonable requests and with approval from the IRB. The data are not publicly available due to their containing information that could compromise the privacy of research participants.
